# Preparation of Lignin-Based High-Ortho Thermoplastic Phenolic Resins and Fibers

**DOI:** 10.3390/molecules26133993

**Published:** 2021-06-30

**Authors:** Yu Ren, Jin Xie, Xiahong He, Rui Shi, Can Liu

**Affiliations:** 1 Key Laboratory for Forest Resources Conservation and Utilization in the Southwest Mountains of China, College of Materials Science and Engineering, Southwest Forestry University, Kunming 650224, China; ry159@outlook.com (Y.R.); Xj920721256@163.com (J.X.); 2 Key Laboratory of State Forestry Administration for Highly-Efficient Utilization of Forestry Biomass Resources in Southwest China, Southwest Forestry University, Kunming 650224, China; hexiahong@hotmail.com

**Keywords:** lignin, thermoplastic phenolic resin, high-ortho, phenolic fiber, thermal stability

## Abstract

Surplus lignin, which is inefficiently used, is generated in the forestry industry. Currently, most studies use lignin instead of phenol to synthesize thermosetting resins which cannot be reprocessed, thus affecting its application field. Thermoplastic phenolic resin has an orderly structure and excellent molding performance, which can greatly improve its application field and economic value. Herein, phenol was partially replaced with enzymolysis lignin (without treatment), generating lignin-based high-ortho thermoplastic phenolic resins (LPRs), and then lignin-based phenolic fibers (LPFs) were prepared by melt spinning. FTIR, ^13^C-NMR and GPC were used to characterize the ortho–para position ratio (O/P value), molecular weight and its distribution (PDI), and rheological properties of the resin. TG, XRD, SEM and tensile property studies were used to determine the thermal stability, orientation, and surface morphology of the fiber. Lignin addition resulted in the decline of the O/P value and molecular weight of the resin. For the 10% LPR, the O/P value, Mw, and PDI were 1.28, 4263, and 2.74, respectively, with the fiber exhibiting relatively good spinnability. The tensile strength and elongation at break of the 10% LPF were 160.9 MPa and 1.9%, respectively. The addition of lignin effectively improved the thermal properties of the fiber, and the carbon yields of 20% LPF before and after curing were 39.7% and 53.6%, respectively, which were 22.2% and 13.7% higher than that of the unmodified fiber, respectively.

## 1. Introduction

In the context of global energy depletion and environmental pollution, research and development regarding biomass energy and materials is particularly critical. In the field of lignocellulosic biomass, cellulose and hemicellulose have been widely studied and applied in the preparations of bioethanol, furan compounds, and carbon-based materials through various biological and chemical pathways [1,2,3]. However, lignin, as the second largest bio-based polymer material and the largest source of natural aromatics, has not been well-utilized and is generally produced as a low-value by-product of cheap energy [4]. Lignin has a phenylpropane structure similar to that of phenol; therefore, it has strong potential to replace phenol in phenolic resin synthesis [5,6]. Pinheiro [7] used the acetic acid solvent method to extract lignin instead of phenol from bagasse at 187 °C and used 95% acetic acid to prepare phenolic resin. Compared with sulfate lignin, these lignins exhibited higher thermal stability, lower molar mass, and lower methoxyl content, and were therefore suitable for phenolic resin synthesis. Wang [8] used a novel solid acid catalytic crystallization process to treat enzymatic hydrolysis lignin (EHL), which reduced the molecular weight of the EHL and improved the phenolic hydroxyl content and reaction activity. The treated lignin replaced 50% of the phenol, generating phenolic foam that showed good heat insulation and compressive strength. Li [9] conducted low-cost depolymerization of alkaline lignin and elucidated the polymerization mechanism. Compared with basic lignin phenolic resin, the phenolic resin prepared using depolymerized lignin cured faster, with good bonding strength. However, there are currently two problems with the application of lignin in phenolic resins: For improvement, lignin has to be further depolymerized, and the procedures are cumbersome and costly. Conversely; the previous research regarding lignin phenolic resin mostly focuses on phenolic foam, adhesives, and other thermosetting resins. The poor processability of the resin limits its applicability [10,11,12].

Thermoplastic phenolic resin displays excellent processability, may considerably improve the applicability and thus the value of the resin, and may be synthesized under acidic conditions. Chemical thermoplastic phenolic resins are widely used in phenolic molding powder, foam plastic, and fiber. Of these, phenolic fiber is a type of 3D cross-linked heat-resistant fiber, which is prepared from thermoplastic phenolic resin by melt spinning and solidifying in a coagulation bath. It was discovered during research into cheap carbon fiber necessary for aerospace applications [13]. Phenolic fiber displays a relatively high limiting oxygen index (34–36%) and excellent flame retardant performance, maintaining the original molecular chain structure after high-temperature combustion. Fabric consisting of phenolic fiber has unique advantages in high-temperature protection, aerospace equipment protection, and other fields [14,15,16,17,18,19,20]. Meanwhile, phenolic fiber is also a precursor in the preparation of activated carbon fiber and carbon fiber/phenolic resin composites, which have been widely used in adsorption and energy storage materials, and in electrochemical fields [20,21]. The wide application of these fibers requires specific parameters of the phenolic resin, such as a narrow molecular weight distribution index and high proportion of linear arrangement. During thermoplastic phenolic resin synthesis, methylene may react at the ortho or para positions on the phenol ring. When more ortho connections occur than para connections (i.e., the O/P value is higher than 1), a high-ortho phenolic resin is formed [22,23]. The high-ortho phenolic resin structure is more ordered, while the highly reactive para-position group is retained, which results in faster curing [24,25,26,27,28,29]. Therefore, the special structure of high-ortho resin is more conducive to phenolic fiber preparation.

In general, the preparation of lignin polymer materials may expand the applicability and considerably improve the economic value of lignin, and numerous studies regarding thermoplastic lignin polymers have been reported [30,31,32,33,34,35,36]. Chen [37] added freeze-dried alkali lignin to polypropylene by melt compounding. The increased interfacial areas and superior dispersity values significantly improved the mechanical properties of the composites, and lignin promoted the ultraviolet and oxidation resistances of the composites. Wang [38] prepared lignin/polylactic acid (PLA) fibers by melt spinning, showing that the introduction of PLA improved the composite spinnability, and the hydrogen bonds formed between the materials improved the tensile modulus of the carbon fiber. Kai [39] prepared lignin-poly (3-hydroxybutyrate) (PHB) composite nanofibers by grafting β-butyrolactone onto lignin. The addition of lignin improved the tensile strength, elongation, and Young’s modulus of the fiber, which exhibited good antioxidant properties and biocompatibility. In the above studies, lignin and thermoplastic polyolefins were blended to prepare lignin thermoplastics. Lignin molecules do not generally react in resin compositions; at the same time, there has been little research regarding lignin thermoplastic phenolic resins. Our team prepared thermoplastic phenolic resin from early-stage lignin liquefaction and discussed its application in fibers [40,41]. In previous studies, lignin liquefaction products were used in resin syntheses. However, high temperatures and strong acids were required for lignin liquefaction, and the preparation steps were complicated, the process was dangerous, and there were various complex problems, such as acid treatment. The process was neither simple nor green. To utilize lignin more efficiently, it is necessary to further simplify the resin synthesis and improve its machinability.

In this study, thermoplastic lignin-based phenolic resin was synthesized by partially replacing phenol with enzymatic hydrolysis lignin, and the phenolic fiber was prepared by melt spinning. The effects of different lignin contents on the resin structure and fiber properties were investigated by adding lignin directly without treatment. The results lay a theoretical foundation for the synthesis of high-performance lignin thermoplastic phenolic resin and efficient lignin utilization.

## 2. Results and Discussion

### 2.1. Characterization of LPRs

#### 2.1.1. FT-IR Characterization of the LPRs

**Figure 1:**
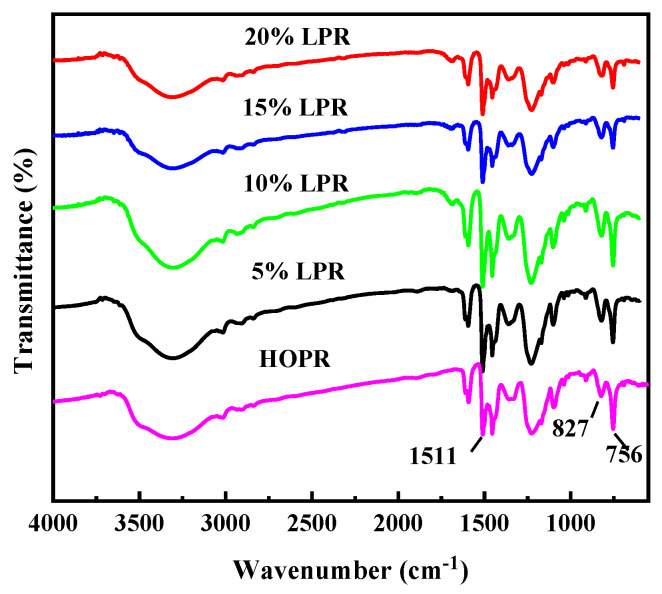
FT-IR spectra of LPRs with different lignin substitution rates.

Figure 1 shows the FT-IR spectra of the LPRs containing different lignin contents. The O–H (PhO-H) of the phenolic hydroxyl group is observed at ~3325 cm^−1^. The stretching vibration of the benzene ring double bonds is observed at ~1593 cm^−1^, and the absorption peak of the benzene ring substituent is observed at ~1511 cm^−1^. With increasing lignin content, the absorption peak intensity increases, because a larger amount of lignin reacts with the benzene ring to form substituent groups. The deformation vibration of the methylene bond (–CH_2_–) is observed at ~1455 cm^−1^, the asymmetric stretching vibration of the ether bond (C–O–C) is observed at ~1100 cm^−1^, and the vibration of C–O(PhCh_2_–OH) in hydroxymethyl phenol is observed at ~1040 cm^−1^. The absorption peak observed at ~827 cm^−1^ represents the para-substituted benzene ring, and at 756 cm^−1^, the ortho-substituted benzene ring. Compared with the IR spectra of high-ortho phenolic resin (HOPR), the characteristic absorption peaks of LPR are almost identical, indicating that the overall phenolic resin structure is largely unaffected by the addition of lignin. However, with increasing lignin content, the ortho-absorption peak observed at 756 cm^−1^ decreases, while the para-absorption peak observed at 827 cm^−1^ increases, showing a decline in ortho-substituted products. The methoxy group of lignin occupies the ortho position relative to the phenolic hydroxyl group, and thus exerts a steric effect on the ortho reaction of the resin, inducing the decrease in the fraction of the ortho product.

#### 2.1.2. ^13^C-NMR Analysis of the LPRs

**Figure 2:**
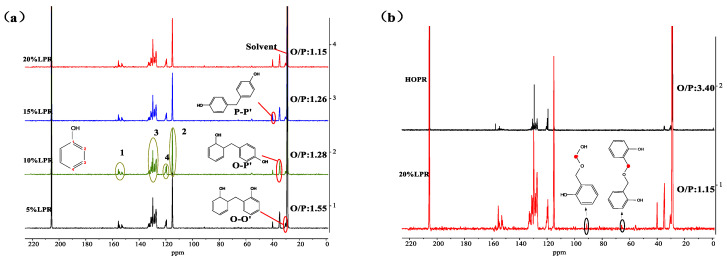
(**a**) The ^13^C-NMR spectra of LPRs with different lignin replacement amounts (5–20%); (**b**) difference of ^13^C-NMR spectra of lignin before and after the resin reaction (20% LPR and HOPR).

To further characterize the ortho/para structures and obtain more accurate O/P values for the lignin phenolic resins, the ^13^C-NMR spectra of the phenolic resins containing different lignin contents are shown in Figure 2. The chemical shift of the solvent, deuterated acetone, is observed at approximately 29 ppm. The signals representing the methylene bonds (–CH_2_–), corresponding to ortho–ortho (O–O’), para–para (P–P’), or ortho–para (O–P’) connections within the phenolic resin, are observed at 31, 40, and 35.5 ppm, respectively. The signals at 155–150 ppm are assigned to C1 connected to the phenolic hydroxyl group, while those observed at 134–126.5 ppm represent ortho- and para-C linked with CH_2_ and the meta position C3 not involved in the reaction. The signals observed at 119–121 and ~115 ppm represent unreacted para-C4 and unreacted ortho-C2, respectively. The three linking modes of methylene (–CH_2_–) are shown in Figure 1. The O/P values of the resins are calculated using the following Equation (1) [42].

(1) $$O/P\;\mathrm{value}=\frac{2\mathrm{AO}-O+\mathrm{AO}-P}{2\mathrm{AP}-P+\mathrm{AO}-P}$$

AO–O’, AO–P’, and AP–P’ are the intensities of the signals representing the O–O’, O–P’, and P–P’ bonds, respectively. The calculated O/P values of the LPRs and HOPR are 1.55, 1.28, 1.26, 1.15, and 3.40. After lignin addition, the O/P ratio of the resin decreases significantly, and with increasing lignin content, the O/P ratio decreases gradually. When the lignin content is 20%, the resin almost no longer displays the characteristics of high-ortho-substitution, which is consistent with FT-IR spectroscopy. The ^13^C-NMR spectra of HOPR and 20% LPR (Figure 2b) suggest that the carbon skeletons of the resins are almost identical after the addition of lignin. However, there are two weak signals observed at ~65 and ~91 ppm for 20% LPR that are not observed in the spectrum of HOPR, and the structure represented by these two signals exhibits a similar linkage to that of carbon atoms within lignin [43]. Hence, lignin is involved in phenolic resin synthesis, generating complex molecular chains, and does not form a simple physical mixture.

#### 2.1.3. Molecular Weights (GPC) and Spinnabilities of the LPRs

**Figure 3:**
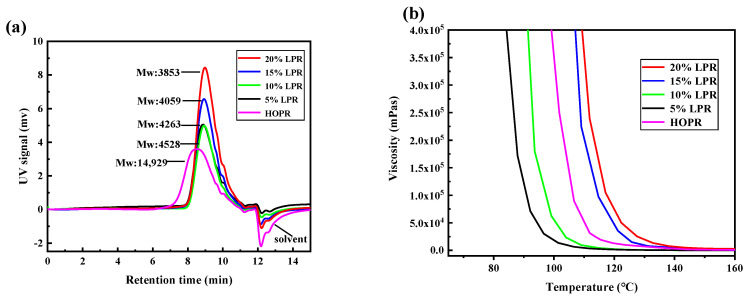
(**a**) GPC chromatograms of LPRs and HOPR; (**b**) the viscosity-temperature curves of LPRs and HOPR.

Figure 3a shows the gel chromatograms of the LPRs containing different lignin contents. The number average molecular weight, weight average molecular weight, molecular weight distribution (PDI) and O/P values of the resins are shown in Table 1. The molecular weight of phenolic resin affects the fiber-forming property and, subsequently, the fiber properties. The larger the molecular weight of the resin, the stronger the thermal and mechanical properties of the resin, and the narrower the PDI, which is conducive to fiber preparation. Compared with that of HOPR, the relative molecular weights of the LPRs are not large, and the average molecular weights of the LPRs decrease with increasing lignin content. This may be because the large amount of methoxyl within lignin causes an increase in the proportion of the monofunctional system within the reaction, which inhibits polycondensation. This terminates molecular chain growth and decreases the formation of the macromolecular chains, thus decreasing the molecular weights of the LPRs. Simultaneously, the system contains more free phenols, and therefore easily generates small molecules, resulting in the reduction in the weight-average molecular weight. This results in an incomplete phenolic reaction and thus facilitates the generation of small molecules within the system, thus broadening the PDI and resulting in a decrease in spinnability. Upon phenolic resin synthesis, the links are not simple and linear, the original linear structure is destroyed, and the spinnability deteriorates, because the lignin does not undergo liquefaction, depolymerization, and other treatments, but maintains the original, more complex, network structure. Among the LPRs, 10% LPR exhibits the narrowest PDI, with a high molecular weight and O/P value and good spinnability relative to those of the other LPRs.

To study the processing properties of the resins and determine the spinning temperatures of the LPRs, rheological studies were performed (Figure 3b). With increasing temperature, the viscosities of the resins decreased sharply, and the curves all exhibited a clear transformation. Above 140 °C, the viscosities of all resins were very low, the resins became fluids, and no longer exhibited spinnability. Prior to fluidization, at the same temperature, the higher the lignin content, the higher the viscosity of the resin, which is negatively correlated with the relative molecular weight of the resin. The structure of lignin itself is complex, and the synthesized LPR does not possess a strong linear structure such as that of regular phenolic resin. When softened using heat, the motion of the molecular segments containing the lignin structure is restricted, resulting in uneven heating of the resin due to poor fluidity, and thus showing higher viscosity at the same temperature. When the lignin content is more than 10%, high viscosity and poor spinnability are still observed when heated to 120 °C. A longer heating time or higher spinning temperature is required to render the resin more fluid, which increases the challenge of the spinning process. Ten percent LPR exhibited a high MW, relatively narrow PDI, high O/P value, and good spinnability at 100–110 °C. Therefore, 10% LPR is the optimal raw material for the preparation of lignin-based phenolic fiber (LPF).

### 2.2. Characterization of the LPFs

#### 2.2.1. TG–DTG Characterization of the LPFs

**Figure 4:**
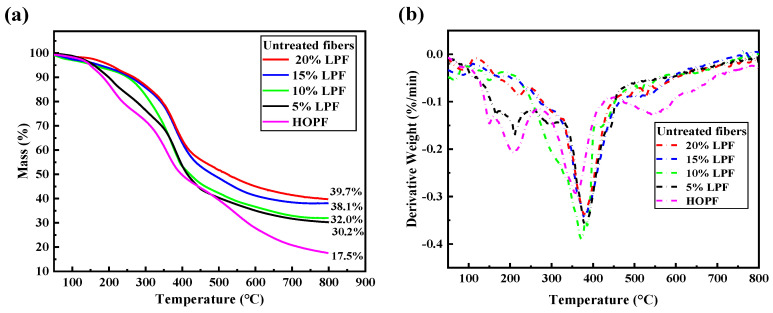
(**a**) TG curves of LPFs and HOPF; (**b**) DTG curves of LPFs and HOPF.

Figure 4 shows the TG–DTG thermograms of the LPFs containing different lignin contents. With increasing temperature, the LPFs maintained continuous mass losses with multiple mass loss steps, which mainly manifested in three stages. In the first stage, at ~100–300 °C, the mass loss is mainly caused by the evaporation of residual water within the resin, the further condensation reaction of the resin, and the decompositions of several small molecules. During this stage, the mass losses of 5% LPF and high-ortho phenolic fiber (HOPF) are clearer, indicating that lignin addition reduces the mass loss here. Lignin was added to the system at the beginning of the reaction and fully mixed with phenol, replacing several small molecules that did not undergo condensation reactions, and thus improving the thermal stability of the resin. Higher lignin content results in a more stable thermogram of the resin. In the second stage, at ~300–450 °C, most of the resin commences decomposition into hydrogen, carbon dioxide, water, and other small molecules, indicated by a large mass loss. During this stage, the highest mass loss is observed, and the rate of mass loss of the LPFs clearly increases, indicating that the thermal stability of LPF is lowest, between 300 and 450 °C. This is caused by the complex structure of lignin disrupting the linear structure of phenolic resin. However, the preparation and uses of the fiber all occur below 300 °C. Therefore, the preparations and properties of the phenolic fibers are largely unaffected. In the third stage, at 450–800 °C, the fiber commences carbonization, with only HOPF exhibiting a relatively clear mass loss during this stage. Due to the complex molecular structure of lignin and the large carbon–carbon bond energy in part of the structure, a higher temperature is required for decomposition, which eventually increases the carbon yield of LPF.

**Figure 5:**
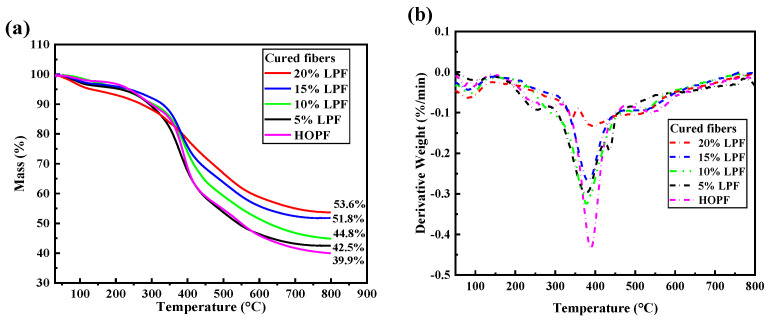
(**a**) TG curves of cured LPFs and HOPF; (**b**) DTG curves of cured LPFs and HOPF.

**Figure 6:**
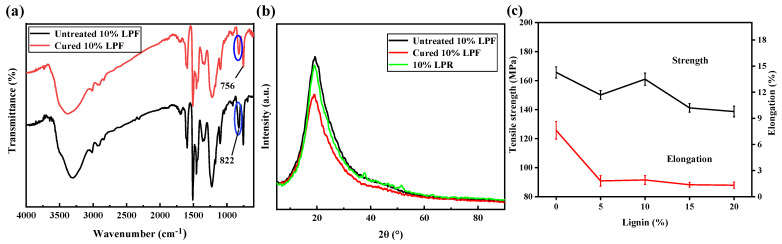
(**a**) FT-IR spectra of 10% LPF before and after curing; (**b**) XRD images of 10% LPR and 10% LPF before and after curing; (**c**) tensile strength and elongation of cured LPFs.

The TG–DTG thermograms of the cured fibers after coagulation-bath and high-temperature treatment are shown in Figure 5. The carbon yields of the cured LPFs were improved, with the carbon yield of the 20% LPF being the highest. In comparison with Figure 4, curing and high-temperature treatment largely eliminated the first-stage mass losses of the fibers and increased their main decomposition stage temperatures. The decomposition stages are not clear until 350 °C, indicating that curing and high-temperature treatment improve the thermal stabilities of the fibers. Similarly, as shown in Figure 5a, lignin addition increases the thermal stabilities of the cured fibers, and the increase in lignin content is positively correlated with the thermal stability, which is consistent with the thermal stabilities of the uncured fibers. Lignin in this study did not undergo additional treatment; therefore, the aromatic structure of lignin was largely retained and the thermal properties in the resin system were improved. According to Figure 6c, the increase in unreactive lignin components within the resin affects the continuity of the resin, leading to a decrease in the tensile strength and elongation at break of the fiber.

#### 2.2.2. Characterization of Structural and Tensile Properties of the LPFs

Figure 6a shows the IR spectra of the 10% LPF before and after curing. The absorption peaks of the main functional groups of the fibers before and after curing are almost identical, because the curing does not involve an additional curing agent (e.g., methenamine), with only formaldehyde solution providing the free hydroxymethyl group to complete the curing, which does not generate a new structure. After curing, the spectrum of the cured fiber still displays strong absorption peaks at 756 cm^−1^ and 822 cm^−1^. Through the integration of the respective peaks and the calculation of the relative O/P values, the O/P value of the fiber before curing is 1.88, whereas the O/P value of the fiber after curing is 1.81. This is because the reactivity of the aromatic ring at the para position of the group is higher, which leads to the relative increase in the intensity of the para position peak and the decrease in the O/P value of the fiber after curing.

In the XRD patterns (Figure 6b), the resin and two fibers exhibit the diffuse fronts of amorphous substances, and no crystal changes occur during the preparation and curing of the fibers. The phenolic resin displays a low-order bond structure, with the structural disorder increasing after lignin doping. Therefore, the preparation of the fiber does not increase the order. During curing, body-shape cross-linking occurs, and the orientation does not change. Therefore, the prepared and cured fibers are amorphous structures.

Figure 6c shows the tensile strengths and elongations at break of the fibers containing different lignin contents. After curing and heat treatment, with increasing lignin content, the tensile strength and elongation at break of the fiber initially increased and then decreased. The homogeneity and continuity of the resin of the blank sample were superior because of the absence of lignin, and the tensile strength of the fiber decreased after the addition of lignin. When the lignin content was 10%, the tensile strength and elongation at break reached the maxima of 160.9 MPa and 1.9%, respectively. In general, the elongation at break did not change much. According to the Mw and PDI data of LPRs, the PDI of 10% LPR was the smallest, while the molecular weight was relatively high. Similarly, according to the DTG data of LPF, the peak of 10% LPF was relatively small, which also indicates the homogeneity of the resin composition. All of these factors had a positive effect on the tensile strength of 10% LPF. The end-capping effect of lignin resulted in the lower molecular weight of the phenolic resin with high lignin content. The complex structure of lignin rendered the molecular chain non-uniform, the PDI was wide, and the fluidity and spinnability of the molecular chain deteriorated. Meanwhile, no modifier was added to the LPF, and the molecular chain structure with higher bond energy was not generated during curing, resulting in poor fracture strength and toughness of the fiber.

#### 2.2.3. SEM Characterization of the LPFs

**Figure 7:**
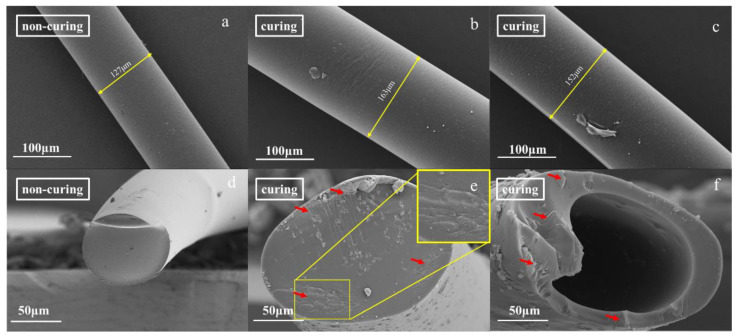
SEM micrographs of the surface and cross section of the 10% LPF and 20% LPF. (**a**,**d**) surface and section of 10% LPF; (**b**,**e**) surface and section of 10% LPF cured fiber; (**c**,**f**) surface and section of 20% LPF cured fiber.

The SEM images of the surfaces and cross sections of the 10% and 20% LPFs are shown in Figure 7. The fiber surfaces were smooth (Figure 7a–c), but there were small amounts of residue, which may have been caused by small-molecule decompositions on the fiber surfaces in strong acid during curing. More surface residue was observed on the cured 20% LPF, which is due to the high lignin content and the small molecular components within the fiber structure, which generate more residues on the fiber surface, resulting in a decrease in the smoothness of the fiber surface. The cross section of the cured 10% LPF (Figure 7e) displays ductile fractures with different fracture directions, indicating that curing and heat treatment improved the degree of cross-linking within the fiber and increased the strength and toughness of the fiber. There are a small number of residues and tiny holes in the cross section, which were due to the volatilization of the chemical reagents dissolved in the fiber and the water generated by the reaction during curing, in addition to the incompletely cured resin structure when the fracture adhesion formed residue. Although the cross section of the cured 20% LPF also exhibited some ductile fracture, it had a core–shell structure. This is due to the lignin complex structure resulting in the slow diffusion of C^+^H_2_OH ions from the fiber surface to the interior. With increasing temperature, the curing rate was accelerated, the outer layer of the fiber gradually solidified, and the movement of C^+^H_2_OH ions was hindered. The internal solidification of the fiber was incomplete, and finally decomposed in the strong acid during curing at high temperature, forming the core–shell structure. This is also the main reason for the lower fracture strength of the 20% LPF relative to that of the 10% LPF. Thus, the replacement amount of lignin should not be too high. When the replacement amount of lignin was 10%, the performance of the LPF was optimal. 

## 3. Materials and Methods

### 3.1. Chemicals

All chemicals used were obtained as follows: zinc acetate, oxalic acid, sulfuric acid, and hydrochloric acid were purchased from Titan Technology (Shanghai, China); ethanol, phenol, and aqueous 37–40% formaldehyde were purchased from Tianjin Zhiyuan Chemical Reagent Co. (Tianjin, China). Lignin, ground to 100–120 mesh, was purchased from Yunnan Yunjinglin Paper (Puer, China). All chemicals were of analytical grade and used without further purification.

### 3.2. Preparation

Phenol and lignin (5, 10, 15, or 20 g) were added to a three-point flask equipped with a reflux condenser, thermometer, and an agitator, and heated at 120 °C for 0.5 h to ensure complete mixing. Subsequently, 4.4 g zinc acetate and 65 g formaldehyde solution were added, and the reaction continued for 3 h. Finally, 2.5 g oxalic acid was added, and the mixture was heated to reflux temperature for 3 h. The mixture was then dissolved in organic solvent and decompressed to remove water and unreacted phenol. After 2 h of precipitation, the LPR was obtained.

LPF was prepared by spinning the modified resin particles in a melt spinning machine at 100–120 °C. Fibrous precursors were soaked in a coagulation bath consisting of 12 wt.% hydrochloric acid and 18.5 wt.% formaldehyde, mixed evenly, and maintained at room temperature for 0.5 h. The curing solution was heated from room temperature to boiling at a heating rate of 20 °C/h and maintained for 1 h. Finally, the cured fibers were further heat-treated at 180 °C for 1 h to complete cross-linking.

### 3.3. Characterization

FT-IR spectroscopy was performed using a Nicolet iS50 FT-IR spectrometer (Thermo Fisher Scientific, Waltham, MA, USA) with the KBr tablet method in the scan range 4000–400 cm^−1^ with 64 scans. Then, ^13^C-NMR spectroscopy was performed using an ECZ 400S NMR instrument (JEOL, Tokyo, Japan). The 50 mg samples were dissolved in deuterated acetone. The methyl peak of deuterated acetone was set to 29 ppm. GPC was conducted using a PL-GPC 50 gel chromatograph (Agilent Technologies, Santa Clara, CA, USA) with 20 mg sample dissolved in *N*,*N*-dimethylformamide, while polystyrene was used as the reference material. The rheological studies were performed using the Haake Mars iQ rotational rheometer (Thermo Fisher Scientific). A 1.5 g powder sample was heated to 180 °C and cooled to 90 °C in steady-state mode to generate the viscosity–temperature curve. TG–DTG was conducted using a TG 209 F3 Tarsus (NETZSCH, Selb, Germany). Samples of 10 mg were heated at 10 °C/min in the temperature range 50–800 °C with nitrogen as the protective gas at a flow rate of 20 mL/min. XRD was performed using an UItima IV X–ray diffractometer (Rigaku, Tokyo, Japan) with the sample scanned in the 2θ range 5–60°. An SU8010 scanning electron microscope (Hitachi, Tokyo, Japan) was used for SEM at an accelerating voltage range of 0.02–30 kV and a working distance of 8.5 mm under vacuum, and the sample was treated with gold spray. The tensile strengths of the fibers were measured using a Baien YG005A electronic single fiber strength machine (Wenzhou, China). The sample was a single fiber 10 cm in length, the tensile speed was 10 mm/min, the pretension was 0.2 N, and the initial tension was 5 N. Each sample was tested 10 times to obtain an average value.

## 4. Conclusions

LPRs containing different lignin contents (5%, 10%, 15%, or 20%) were prepared by the partial substitution of phenol through direct addition, and LPFs were then successfully prepared. Structural analyses of the prepared LPRs showed that lignin participated in the resin reaction and did not form a simple physical mixture. Lignin addition resulted in a decrease in the O/P value, and the complex structure and sealing effect of lignin resulted in a decrease in the molecular weight and an increase in the PDI. For example, the molecular weight of the 10% LPR was 4263, the PDI was 2.74, and the spinning temperature was 100–110 °C with good spinnability. Lignin improved the thermal stability of the fiber, and the carbon yields of the 20% LPF before and after curing were 39.7% and 53.6%, respectively, which were 22.2% and 13.7% higher than those of unmodified fiber, respectively. Lignin had a considerable influence on the fiber morphology, with residue observed on the fiber surface and micro-holes in the cross section. When the replacement amount of lignin was 20%, a core–shell structure was observed, which reduced the fiber strength and toughness. The fiber morphology and tensile properties of the 10% LPF were optimal, with a tensile strength of 160.9 MPa and an elongation at break of 1.9%. In our previous work, phenolic fibers were prepared using lignin liquefaction [41]. The O/P values of the LPRs were lower than those of the resins prepared using liquefaction, and the average molecular weights of the LPRs were smaller. Due to the complex structure of lignin, the resin synthesized directly using lignin exhibited poor continuity, the core-shell structure was observed in the cured fiber, and the tensile strength of the fiber was relatively low. Although the fiber prepared using liquefaction was superior, the direct addition of lignin considerably reduces the preparation complexity and costs and considerably improves the possibility of using LPRs for industrial applications.

## Figures and Tables

**Table 1:** O/P value, PDI and molecular weight of LPRs and HOPR.

Sample	O/P Value	Mn	Mw	PDI
0%	3.40	12,736	14,929	1.17
5%	1.55	1350	4528	3.36
10%	1.28	1553	4263	2.74
15%	1.26	1324	4059	3.06
20%	1.15	1373	3853	2.80

## Data Availability

The data presented in this study are available on request from the corresponding author.

## Sample Availability

Samples of the compounds are available from the authors.
